# Widespread seawater intrusions beneath the grounded ice of Thwaites Glacier, West Antarctica

**DOI:** 10.1073/pnas.2404766121

**Published:** 2024-05-20

**Authors:** Eric Rignot, Enrico Ciracì, Bernd Scheuchl, Valentyn Tolpekin, Michael Wollersheim, Christine Dow

**Affiliations:** ^a^Department of Earth System Science, University of California, Irvine, CA 92697; ^b^Radar Science and Engineering Section, Jet Propulsion Laboratory, California Institute of Technology, Pasadena, CA 91109; ^c^Department of Civil and Environmental Engineering, University of California, Irvine, CA 92697; ^d^ICEYE Oy, 02150 Espoo, Uusimaa 02150, Finland; ^e^Department of Geography and Environmental Management, University of Waterloo, Waterloo, ON N2L 3G1, Canada

**Keywords:** interferometry, Antarctica, sea level rise, Southern Ocean, hydrology

## Abstract

Warm water from the Southern Ocean has a dominant impact on the evolution of Antarctic glaciers and in turn on their contribution to sea level rise. Using a continuous time series of daily-repeat satellite synthetic-aperture radar interferometry data from the ICEYE constellation collected in March–June 2023, we document an ice grounding zone, or region of tidally controlled migration of the transition boundary between grounded ice and ice afloat in the ocean, at the main trunk of Thwaites Glacier, West Antarctica, a strong contributor to sea level rise with an ice volume equivalent to a 0.6-m global sea level rise. The ice grounding zone is 6 km wide in the central part of Thwaites with shallow bed slopes, and 2 km wide along its flanks with steep basal slopes. We additionally detect irregular seawater intrusions, 5 to 10 cm in thickness, extending another 6 km upstream, at high tide, in a bed depression located beyond a bedrock ridge that impedes the glacier retreat. Seawater intrusions align well with regions predicted by the GlaDS subglacial water model to host a high-pressure distributed subglacial hydrology system in between lower-pressure subglacial channels. Pressurized seawater intrusions will induce vigorous melt of grounded ice over kilometers, making the glacier more vulnerable to ocean warming, and increasing the projections of ice mass loss. Kilometer-wide, widespread seawater intrusion beneath grounded ice may be the missing link between the rapid, past, and present changes in ice sheet mass and the slower changes replicated by ice sheet models.

## Significance

We present evidence for seawater intrusions occurring at tidal frequencies over many kilometers beneath the grounded ice of Thwaites Glacier, West Antarctica, a major contributor to sea level rise. The results call into question the traditional approach of modeling a fixed, abrupt transition from grounded ice to ice floating in the ocean with no ice melt at the transition boundary. We delineate a tidally controlled grounding zone, 2 to 6 km in length, and additionally irregular seawater intrusions extending another 6 km inland at spring tide. The rushing of seawater beneath grounded ice over considerable distances makes the glacier more vulnerable to melting from a warmer ocean than anticipated, which in turn will increase projections of ice mass loss.

The Antarctic Ice Sheet has been a major contributor to sea level rise over the past four decades (1). The mass loss is not caused by a decrease in snowfall, but by a speed-up of glaciers in West Antarctica, the Antarctic Peninsula, and the Wilkes Land sector of East Antarctica. Glacier speed-up has been attributed to an increase in glacier melt in contact with warm, salty ocean waters of circumpolar origin, or circumpolar deep water (CDW) (2). CDW has gained access to the continental shelf, ice cavities, and glaciers over the past 40 y (3) due to an increase in the strength of the westerly winds (4), itself caused by the combined effect of rapid climate warming over the rest of the planet from human-induced greenhouse gas emissions and a cooling of the Antarctic stratosphere from the human-induced depletion of the stratospheric ozone (5, 6).

**Fig. 1.**
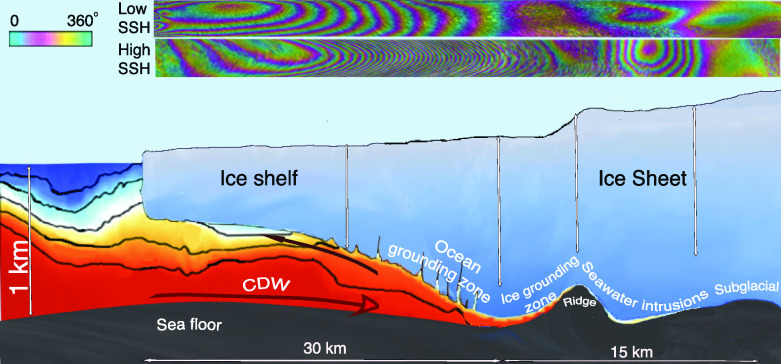
Ocean grounding zone vs. ice grounding zone of an ice sheet/ice shelf system. CDW filling the ice cavity is color coded by temperature from blue (cold) to red (warm). The ocean grounding zone is always flooded with CDW. The ice grounding zone alternates between flooded and unflooded with changes in oceanic tide and atmospheric pressure. Seawater intrusion propagates beyond the ice grounding zone at irregular intervals. On grounded ice, the glacier bed is overlaid by a thin sheet (10 cm) of pressurized subglacial water, which facilitates intrusion and hydraulic jacking of seawater. The panel on the *Top* shows differential, interferometric fringes associated with ice flexing in the ocean and ice grounding zones at low (*Top*) and high sea surface height (SSH) (*Below*). Bull’s eye deformation fringes farther upstream reflect ice subsidence vs. uplift around a bed depression at low vs. high SSH.

Central to the glacier loss are the physical processes taking place at the transition boundary between grounded ice and ice floating into the ocean, or “grounding line.” At the grounding line, the glacier traverses hydrostatic equilibrium (HE), then becomes depressed a few meters below HE in the flexure zone, before achieving full HE another O(10 km) (O(): abbrev. of the order of) downstream (7) (Fig. 1). Ice melts vigorously at the ice base when first in contact with seawater because the freezing point of the ice/seawater mixture is −1.9^°^C at the surface vs. 0^°^C for freshwater, and decreases by 0.75^°^C for every kilometer of water depth, i.e., is −2.7^°^C at 1,100 m depth. CDW at +1 to +2^°^C therefore has a thermal forcing of 3.7 to 4.7^°^C that will vigorously melt ice at depth. Melting of grounded ice will remove basal resistance to glacier flow over a length, *L*, O(1 km), multiplied by the glacier width, *W*, O(20 km), i.e., an area O(20 km^2^) which offers basal resistance to flow (8).

Locating the grounding line is done most precisely using double difference satellite radar interferometry, or Differential Interometric Synthetic Aperture Radar (DInSAR) (9–13). Here, we use a dense time series of satellite InSAR observations acquired in Spring 2023 by the U.S./Finnish ICEYE Synthetic Aperture Radar (SAR) constellation, at the X-band frequency (9.65 GHz or 3.10 cm wavelength). ICEYE operated two satellites on a 1-d ground track repeat, daily, in March–June 2023 (14). The ICEYE DInSAR data document the short-term migration of the grounding line at an unprecedented level of detail, at a daily resolution, across the core of faster flow of Thwaites Glacier, or “main trunk” (*Materials and Methods*). We define the envelope of grounding line locations revealed by DInSAR over multiple tidal cycles as “ice grounding zone,” as elaborated below.

In 2018, a portion of the ice grounding zone mapped with Cosmo-SkyMed (CSK) data (X-band frequency, 1-d repeat acquired every 16 d) abutted a bedrock ridge at 900 m depth, hereby named “Mouginot Ridge,” approximately 5 km in length (along flow), 9 km in width (across flow), and 200 m in height according to BedMachine Version 3 (15) (Fig. 1). Mouginot Ridge provides a temporary barrier for the glacier retreat as its prograde bed slope (bed elevation rises in the inland direction) protects the glacier from a marine ice sheet instability (16). Another 13 km inland is a second ridge, hereby named “South Ridge,” approximately 5 km in length (along flow), 5 km wide (across flow), at 960 m depth, before bed elevation drops below 1,000 m depth over the next 17 km, and then below 1,300 m another 30 km farther upstream (Fig. 1). At that point, the bed elevation drops into the deeper Bentley Subglacial Trough, and the glacier retreat will accelerate and may not stop until the entire basin drains to sea (17). Subsequent to that, other marine-based parts of West Antarctica will collapse for a combined total sea level rise of 3 m. Understanding how long it takes for Thwaites Glacier to retreat past Mouginot and South Ridges is therefore critical to the future of Thwaites, West Antarctica, and in turn global sea level rise.

At present, Thwaites Glacier drains an area of 192,760 km^2^, with an ice volume above sea level that is equivalent to a 65-cm global sea level rise (SLR), and a cumulative mass loss to the ocean of 634 Gt during 1979 to 2017 (1 Gt = 10^12^ kg) (1). During 1992 to 2011, the grounding line retreated at 1 km/y at the center, one of the fastest retreat rates in Antarctica (16). Between 2011 and 2017, the retreat rate decreased by about half (18).

Many ice sheet models assuming zero melt at a fixed grounding line have difficulties replicating the ongoing retreat of Thwaites (7, 19). An efficient and necessary control on glacial retreat for these models is to collapse a buttressing ice shelf. Thwaites Ice Tongue however offers little to no resistance to flow (20). Simulations of the unpinning of the Eastern ice shelf indicate little impact on the system (7, 21, 22). The dominant physical process that drives the glacier retreat is therefore missing from these models.

Recent satellite data suggest that the transition boundary between grounded and floating ice ought to be represented as a “grounding zone” instead of a grounding line (Fig. 1). The grounding zone length, *L*, is larger than anticipated from HE, i.e., O(1 km) instead of O(100 m) (18, 23) due to the need to update the bed topography. A comprehensive survey of Antarctica grounding lines using a machine learning algorithm confirms that km-wide ice grounding zones are widespread (13, 24, 25). The ice grounding zone is the region where the grounding line migrates over the tidal cycle (Fig. 1). It differs from the “ocean grounding zone” where ice experiences tidal flexure and is flooded with seawater (26). The ocean grounding zone has been documented using other techniques, e.g., laser altimetry (27). The ice grounding zone is the focus of this study. To observe it, we use a unique time series of daily, high-resolution satellite observations of the main trunk of Thwaites Glacier (14).

## Results

**Fig. 2.**
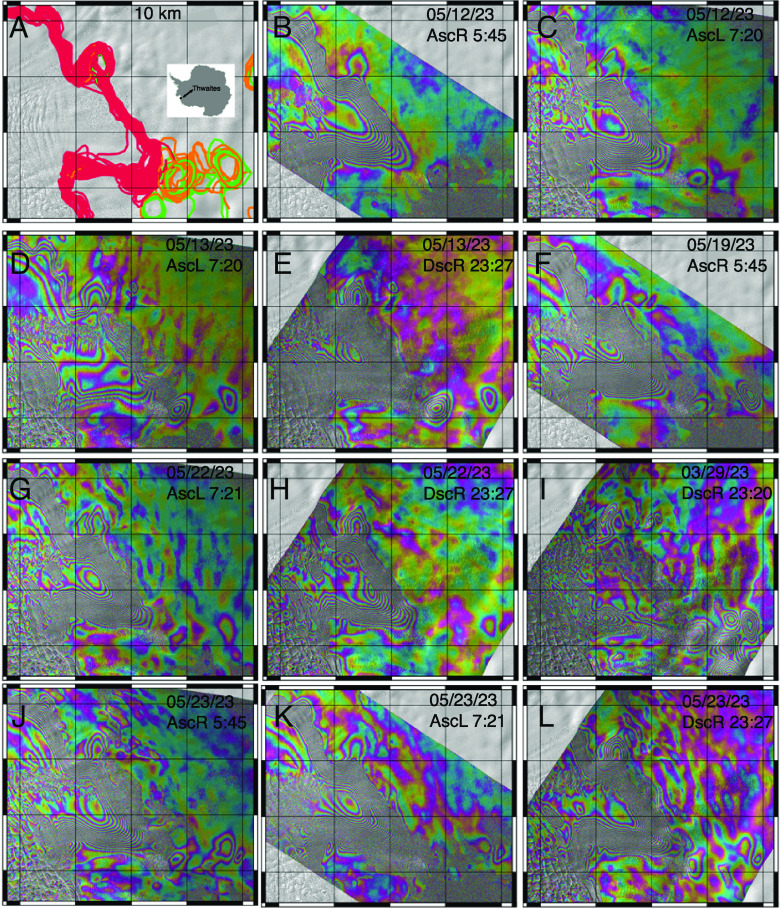
Grounding line positions derived from ICEYE DInSAR on the main trunk of Thwaites Glacier, West Antarctica, in March–June 2023. (*A*) Red lines delimit regions moving in phase with changes in SSH, or ice grounding zone. Orange lines delineate irregular seawater intrusion moving in phase with SSH, whereas green lines delineate seawater extrusion out of phase with SSH. (*B*–*L*) Example differential interferogram (DInSAR) with Ascending Right (AscR), Ascending Left (AscL) and Descending Right (DscR) looking geometry and Descending (Dsc) right looking geometry at different UTC time (e.g., *B*) is 5:45am UTC). Each fringe cycle is a 360^°^ change in phase, equivalent to a 1.65 cm displacement in line-of-sight distance of the ice surface. The incidence angle is 12, 18, and 34^°^ for DscR, AscL, and AscR. Gray background is a shade relief of the surface topography.

ICEYE imaged Thwaites Glacier along a descending right-looking track and two ascending left- and right-looking tracks (Fig. 2). Ascending left and right tracks were acquired two hours apart, at 5 am and 7 am UTC, respectively. Descending right tracks were acquired another 6 h later, at 11 pm UTC. With the DInSAR technique, we measure a differential vertical motion of the ice surface caused by tide-related changes in SSH over three epochs, not an absolute tidal position of the grounding line (*Materials and Methods*) (9).

### 1.1. Tidally Driven Changes in SSH.

**Fig. 3.**
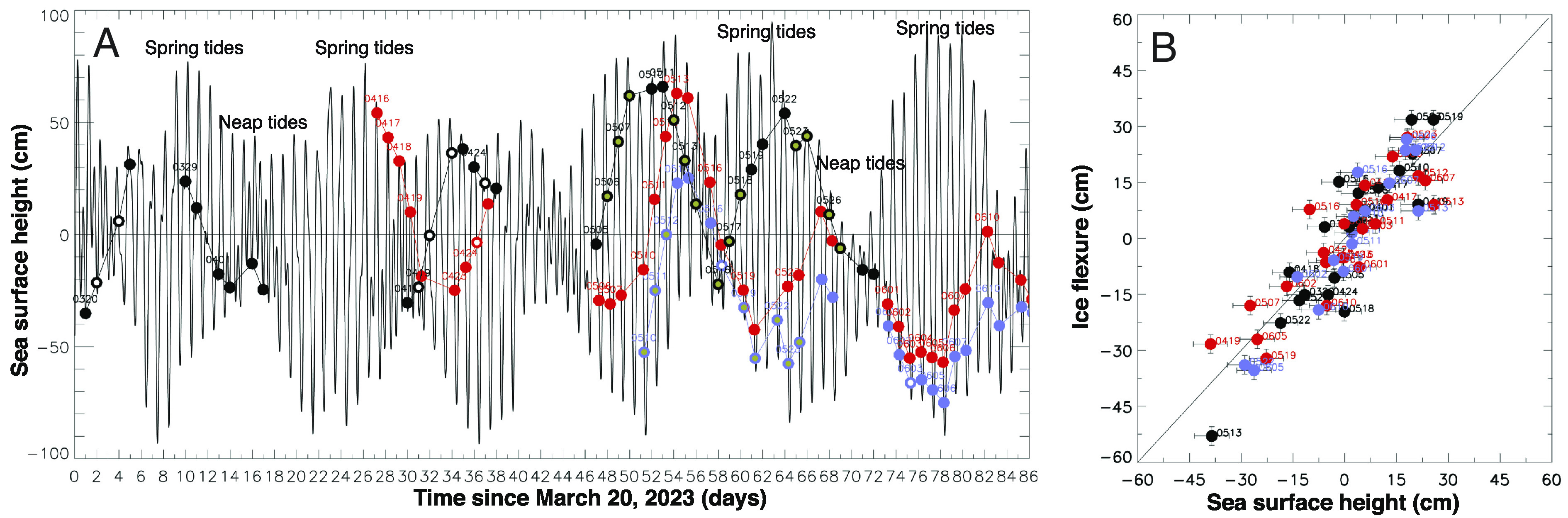
Modeled vs. observed changes in SSH (cm) for Thwaites Glacier, Antarctica. (*A*) time series of SSH using the CATS2008 tidal model (28) corrected for the Inverse Barometric Effect (IBE) using ERA-5 atmospheric pressure fields (thin black) vs. time of passage of ICEYE for 3 (or 4) ascending right (red, 5 am UTC), ascending left (blue, 7 am UTC), and descending right (black, 11 pm UTC) tracks. When not occluded by data noise, seawater intrusions are noted with a yellow inner circle vs. no intrusion as white inner circles. (*B*) Comparison of differential changes in SSH from CATS2008 and IBE vs. flexure (in centimeters) measured by DInSAR across the ocean grounding zone with error bars.

At a 1-d repeat, the ocean tidal state is dominated by the solar cycle and drifts only slowly every 24 h. SSH is also modulated by changes in barometric pressure, or inverse barometer effect (IBE), at a rate of 1 cm per millibar of change in pressure. Air pressure varies more slowly than the tidal cycle but modulates SSH significantly. We verify that the observed vertical motion in the satellite data agrees well with tides corrected for IBE (*Materials and Methods*) (0 mean with *σ* = 8 cm in Fig. 3).

### 1.2. Migration with Changes in SSH.

Interferograms acquired two hours apart (Fig. 2*B* & *C* and *J* & *K*) reveal a slow change in the grounding line position, as expected for a small change in SSH. In data acquired 6 h later (Fig. 2*D* & *E*, *G* & *H*, and *K* & *L*), SSH changes completely (Fig. 3*A*), and the tidal flexure changes sign. In several DInSAR scenes, the differential motion signal is nearly zero (Fig. 2*I*) and the grounding line is difficult to delineate. Conversely, at the top of the tidal cycle, e.g., at spring tide (sun and moon combine to yield the highest gravitational pull), the differential SSH signal is large, and the limit of tidal flexing is well defined. At neap tide (sun and moon at right angles combine to yield the lower gravitational pull), seawater intrusions are absent (Fig. 2*G*–*K*). At spring tide (Fig. 2*B*–*F*), seawater intrusions prevail.

### 1.3. Ice Grounding Zone Length.

The ice grounding zone is 2 km wide on the east and west flanks of the main trunk, and 6 km wide in the central part. The ice grounding zone is narrowest along steep, prograde bed slopes and widest along shallow bed slopes. A similar sensitivity to bed slopes was found on Amery Ice Shelf (24). In several DInSAR scenes (e.g., Fig. 2*D*, *H*, and *L*), the ice grounding zone extends laterally, e.g., on the west flank of Mouginot Ridge, where the bed is deeper, indicating preferential migration along shallow bed slopes.

With the 2018 CSK data, we could not detect the grounding zone on the east and west flanks of Thwaites due to high rates of ice deformation (18). The precision of determination of the grounding zone was less because data noise in the CSK data was higher. The higher-quality ICEYE data benefit from: 1) a finer spatial resolution: 0.75 m in range for ICEYE vs. 2.1 m for CSK, i.e., a factor 3; 2) a shorter interferometric baseline between successive passes for ICEYE, which offers better phase coherence; and 3) a continuous coverage at daily frequencies with ICEYE which provides a smoother change in environmental conditions from one pass to the next, hence better signal coherence.

### 1.4. Grounding Zone Retreat Rate.

When comparing the ICEYE results from year 2023 with prior ice grounding zone mappings in 2016 to 2018, we find a 3.5 ± 1 km retreat at the glacier center, i.e., a retreat rate of about 0.5 km/y consistent with the 0.6 km/y recorded in 2011 to 2018 (18), and half of the rate (1km/y) observed in 1992 to 2011 (16).

### 1.5. Seawater Intrusion.

**Fig. 4.**
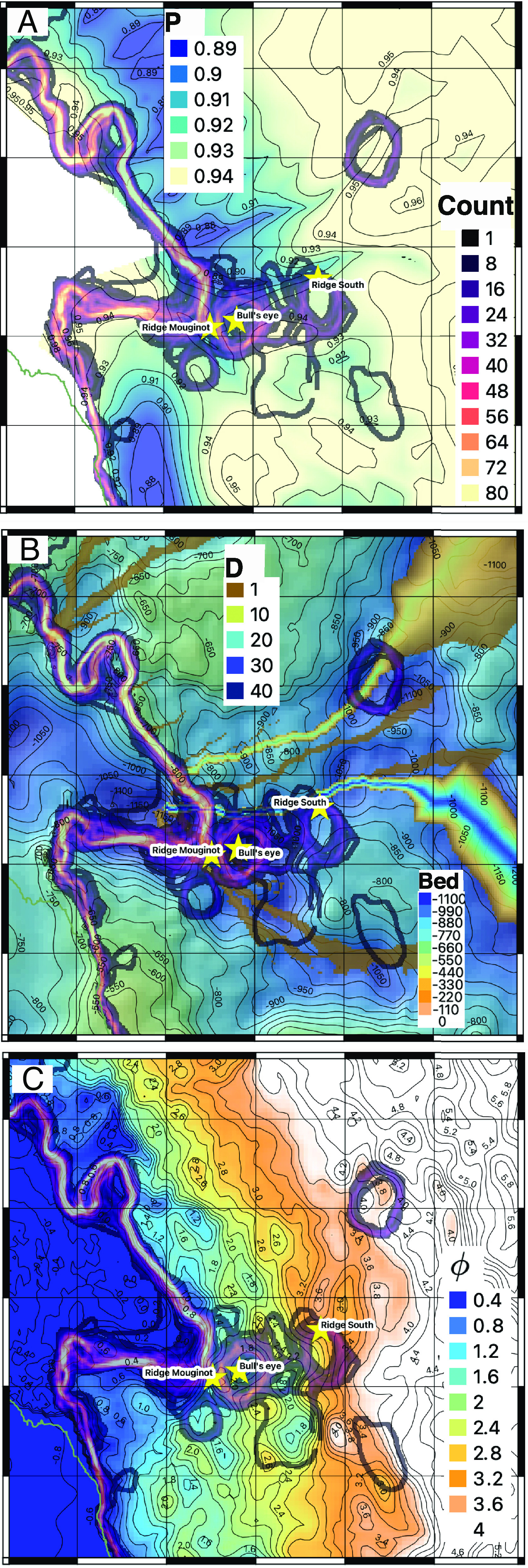
Ice Grounding Zone vs. GlaDS subglacial hydrology. Counts of grounding line position color coded from black (low, 1) to purple and yellow (high, 80) overlaid on (*A*) water pressure, *P*, as as fraction of overburden pressure (0.89 to 0.94) with 0.01 contours levels; (*B*) water discharge, *D*, from 0 to 40 m^3^/s, overlaid on bed topography from 0 to 1,100 m with 50-m contour levels; and (*C*) hydraulic potential, *ϕ*, from 0.4 to 4.0 MPa (Mega Pascal) with 0.2 MPa contour levels; along with location of “Ridge Mouginot” and “Ridge South” ridges, and "Bull’s eye.” The regions of widest intrusions are high-water pressure zones. The subglacial water channels are in low pressure zones.

Along the main trunk of Thwaites, we detect vertical motion beyond the ice grounding zone, on an irregular basis (e.g., Fig. 2 *G* and *H*), that forms a bull’s eye (*Materials and Methods*). We attribute the bull’s eye to irregular “seawater intrusion” that occurs on an irregular basis, aka extreme events. In most cases, the bull’s eye moves in phase with the change in SSH, hence consistent with seawater rushing beneath grounded ice and lifting it up. The intrusions peak in a bed depression revealed by the extensive, precise, and dense (1.5 km) bed mapping performed by NASA’s Operation IceBridge in 2002 to 2019 (29). In other cases, the seawater intrusions are out of phase with SSH, hence indicating seawater trapped in the cavity and/or being flushed out. The magnitude of the vertical motion during extrusions is the same as for intrusions. The envelope of intrusion and extrusion follows a well-defined pattern aligned with the topographic depression upstream of Mouginot Ridge (Fig. 4).

A second persistent bull’s eye is found on the northern flank of South Ridge. We detect no vertical motion between the two bull’s eyes, except in a few scenes acquired in late March, with low differential SSH, at spring tide (Fig. 2*I*). The two bull’s eyes are disconnected. In contrast, the ice grounding zone and the first bull’s eye are so intertwined that it is often difficult to define the exact transition boundary (Fig. 2*A*). The bull’s eye on the South Ridge is less active.

In 2023, we have 58 independent grounding lines. Data quality is sufficient to detect the full extent of seawater intrusion in 33 of them. Among these 33 cases, we detect intrusion (i.e., more than one fringe of deformation) in 16 cases, small intrusion (one fringe or less) in 9 cases, and no intrusion in 8 cases, or 25%. We find a strong correlation between SSH at spring tide and the length of intrusion, i.e., we detect longer intrusions when the acquisitions include a large, positive, spring tide (Fig. 3a). Conversely, at neap tide (e.g., April 2023), i.e., low positive SSH and low probability of seawater intrusion, we find less activity upstream of the ice grounding zone (Fig. 3*A*).

When examining DInSAR scenes acquired the same day at 5am, 7 am, and 11 pm, seawater intrusions are either present or absent the entire time, hence persist on time scales of hours. In contrast, in the bull’s eyes farther upstream, the signal persists for days and subsequently disappears for days (Fig. 2), i.e., has a different temporal dynamics.

### 1.6. Hydraulic Potential.

The hydraulic potential, *ϕ*, controls the mobility of subglacial water beneath the ice sheet. *ϕ* reaches 1.9 MPa at the top of Mouginot Ridge and decreases to 1.5 MPa at the center of the first depression, similar to the level reached about 3.5 km downstream in the ice grounding zone (Fig. 4*C*). Water accumulating in the first bull’s eye is therefore likely seawater that contours Mouginot Ridge. In contrast, the hydraulic potential at the South Ridge is 2.5 MPa and farther upstream, in the next area of water accumulation/drainage, *ϕ* is 4 MPa. Given a change in pressure of 10 kPa for every meter of water, this difference in hydraulic potential is equivalent to O(100 m) of water. We conclude that the bull’s eyes at South Ridge and the ones upstream are caused by subglacial water, not by seawater.

### 1.7. Subglacial Hydrology.

We compare the location of seawater intrusions in the DInSAR data with the modeled subglacial hydrology from the Glacier drainage system model (GlaDS) (30, 31). GlaDS calculates subglacial water production, water discharge, and water pressure beneath Thwaites and allows coincident development of both distributed and channelized systems (*Materials and Methods*). GlaDS predicts an average water thickness of 8 ± 1 cm in the main trunk of Thwaites, with little spatial variability, and a set of subglacial channels aligned with bed troughs, with discharges up to 40 m^3^/s (Fig. 4*B*). The bull’s eye of seawater intrusions near the ice grounding zone aligns between two GlaDs channels over an area of high water pressure, i.e., at 94% the ice overburden pressure (Fig. 4*A*). Subglacial channels are predicted in narrow zones of lower water pressure (91%), coincident with bed troughs.

## Discussion

### 2.1. Ice Grounding Zone Length.

is one order of magnitude larger than that predicted from HE, i.e., bed and surface slope. If we assume that ice deviates from flotation by a few meters due to bending stresses (26), along with uncertainties in ice elevation, our observations are consistent with a proximity to HE.

### 2.2. Interpretation of Seawater Intrusions.

In the bull’s eye above the ice grounding zone, we attribute flexing to seawater intrusions. We are not aware of other physical processes that explain this motion. Tidal bending about a “fixed” grounding line acting like a fulcrum (32) would induce a small-amplitude flexing O(1 cm) of the opposite sign with the tide, which is not the case. We observe flexing upstream occurring both in phase and out of phase with the tide, and the amplitude varies with time and often exceeds 10 cm. These variations are caused by seawater intrusions when in phase with SSH, and extrusions or water being trapped when out of phase with SSH, which occur, respectively, at high and low SSH. Similarly, the motion recorded above the ice grounding zone is not caused by a horizontal motion of the zone of deflation as in ref. 10 because that signal is small and propagates only over distances of a few hundred meters.

Seawater intrusion in the presence of a subglacial freshwater wedge was discussed by Wilson et al. (33) without tides. In their theory, seawater transport is a diffusive process driven by differences in density. Here, the driving mechanism for the intrusions is a change in water pressure that is sufficient to jack up the ice surface at high tide. The two approaches are different and could occur together. Our observations do not help constrain the diffusive process.

### 2.3. Rushing of Seawater.

Ice flexing in the main trunk has a range of ±70 cm vs. a subsidence/uplift of O(10 cm) upstream, i.e., seawater intrusions are thin. For seawater to rush multiple kilometers in half the diurnal cycle, or 6 h, requires speeds O(10cm/s). For a 6-km intrusion in 6 h, the water speed is 28 cm/s. At the upper part of the tidal cycle, we detect intrusions of 12 km inland, which imply a water speed of 56 cm/s. In the “shallow water” assumption, the wave speed will be $\sqrt{gH_{w}}$, where *g* is the acceleration of gravity and $H_{w}$ is the water thickness, or O(1 m/s) for $H_{w}$ O(10 cm). During a transition to low tide, seawater extrusions may be limited by the resistance to water flow in the cavity (34) or be trapped in bed pockets (35).

### 2.4. Implications for Ice Melt.

For a tidal amplitude of ±1 m, the ice grounding zone cavity will fluctuate from 0 to 2 m at the entrance and taper linearly down to zero at the end, i.e., an average water thickness $H_{w}=1$ m. The ice grounding zone of the main trunk is 210 ± 30 km^2^ in size. The water volume will change by 0.21 km^3^ in 6 h at a density of 1,028 kg/m^3^. With a seawater temperature, *T* = 1.0 ^°^C (36, 37) and a freezing point of seawater, *T_f_* = −2.65°C at 1,100 m depth, a salinity of S=35 psu, the ocean thermal forcing, $\left(T-T_{f}\right)$, is +3.65^°^C. Using a heat capacity of cold seawater, $C_{p}=$3,974 J kg^−1 °^C^−1^, converting water volume to mass (1,028 kg per cubic meter), *m*, we obtain an ocean heat transfer, $Q=mC_{p}\left(T-T_{f}\right)$ of 1.5 10^11^ W to the ice. If all the ocean heat were available to melt ice with a latent heat of fusion, $L_{f}=$ 334,000 J/kg, which is a maximum rate of efficiency, we would have a maximum freshwater flux production of 43 mSv (1 Sverdrup = 1 million cubic meter per second), or an average melt rate of 65 m/y within the ice grounding zone. More likely, the heat intrusion is less effective at melting the ice and the melt rate is lower. Similarly, a melting of 3 cm of ice (which has an effective temperature of −90^°^C) would cool the 1-m column of water by 3^°^C and nullify the thermal forcing but still results in melt rates of 20 m/y. In the bull’s eye zone, the intrusions are less regular, and the incoming water will be cooler, so the melt rates should be much lower.

### 2.5. Impact of the Subglacial Water System.

Zones of seawater intrusion focus between the channels, in areas of high water pressure, which makes it easier to lift up the ice surface as observed with DInSAR. If seawater intrusions occur in subglacial water channels, the pressure change may not be sufficient to cause ice uplift. If seawater intrusions occurred only in channels, the ice deformation would align with the channels, which is not what we observe.

Farther inland, beyond the South Ridge, the bull’s eyes line up with a subglacial channel. We attribute its presence to subglacial water flowing from one accumulation pocket to another one (38). The subsidence or uplift of the surface persists for days. For instance, we detect surface uplift of a cumulative 23 cm between 03/29 and 04/05 in the descending right track and a cumulative subsidence of a similar amount from 05/13 to 05/26 consistently in all three tracks, hence a filling event followed by an emptying. In the third series of bull’s eyes, 17 km south of South Ridge, we detect a regular deformation activity in the descending right tracks, with subsidence side by side with uplift (Fig. 4).

The subglacial hydrology system in Antarctica is not a seasonal system that develops during summer melt and shuts down during the winter freeze like in Greenland, but a system at near-steady state (31). Because of the lack of seasonality, the subglacial system is highly pressurized (Fig. 4), which facilitates seawater intrusion and hydraulic jacking at high SSH.

### 2.6. Implications for Ice Sheet Modeling.

Prior work has evaluated the impact of kilometer-wide ice grounding zones on glacier flow. The authors used indirect evidence for the presence of seawater at the glacier base from bright radar echo reflections, typical of a wet and smooth salty bed, extending kilometers inland of a “fixed” grounding line before gradually fading to weaker echoes typical of ice over a thin layer of freshwater (39). With km-scale intrusions, ice sheet models project larger rates of mass loss (39–41). Such widespread intrusions have been indirectly suggested near grounding lines, over large regions, instead of narrow channels (42). Despite these pioneer studies, the physical processes driving melt under grounded ice have not been included in ice sheet models, due to a lack of more direct evidence for such intrusion (43).

Our results confirm the existence of kilometer-size grounding zones on the main trunk of Thwaites Glacier. Models with km-size grounding zones and vigorous ice melt will produce higher projections of glacier loss, possibly by a factor of 2 (32, 39, 41, 44). The corresponding increase in ice sheet sensitivity to ocean warming may explain the inability of models at reproducing rapid rates of sea level rise during past warm periods (19) or during the recent past (45).

### 2.7. Future Retreat.

At present, the GZ of Thwaites Glacier is retreating along a prograde slope, which is a configuration conducive to stabilization. Indeed, the grounding line retreat rate is twice slower than in the past when the grounding line was retreating on a flat bed (Fig. 2). Yet, the retreat is not halted by the prograde slope. Seawater intrusions extending 12 km may set up the glacier for further retreat (17). Once the glacier grounding zone retreats past Mouginot Ridge, which could happen in the next few years, it will migrate quickly to the South Ridge along retrograde slopes. South Ridge will be a smaller obstacle. It may only take 10 to 20 y before the glacier retreats past South Ridge, into the deeper basin, at which point a fast retreat will resume. In the nearby smaller-size basins of Smith and Pope Glaciers, retreat along retrograde slopes has proceeded at 2 to 3 km/y for multiple years (46). We conclude that the future of Thwaites—and other Antarctic glaciers—will hinge on how fast warm waters erode grounding zones over large distances, much faster than anticipated by current models.

## Materials and Methods

### Differential Interferometry.

Differential interferometry measures a differential change in ice displacement between 3 (or 4) epochs after eliminating the horizontal motion of ice through differencing of two pairs: we subtract two consecutive 1-d pairs to eliminate the (steady) horizontal motion of the ice and leave the short term (vertical) motion of the ice, i.e., the vertical motion caused by changes in oceanic tide and atmospheric pressure, plus noise. If there is a data gap in data acquisition, we combine 2 × 1-d pairs acquired more than 1 d apart, hence four scenes. Few DInSAR pairs of Thwaites have been available in the past: 2 × DInSAR pairs in 1992 from the Earth Remote Sensing satellite (ERS-1, C-band frequency, 5.6 GHz or 5.6 cm wavelength), 1 × DInSAR pair in 1994 from ERS-1, and 2 × DInSAR pairs in 1996 from ERS-1/2 to map the grounding line and report a retreat of 1 km/y (20). The ERS-1/2 data were not sufficient to characterize the short-term variability in the grounding line position. No data were acquired until 2011 with 2 × DInSAR pairs at the conclusion of the ERS-2 mission (16). Other SAR satellites, e.g., the European Space Agency (ESA) Envisat ASAR and Sentinel-1 (5.5 GHz or 5.7 cm wavelength), the Canadian Space Agency (CSA) RADARSAT-1 and RADARSAT-2 (ditto), the Japanese Space Agency (JAXA) PALSAR (L-band, 1.2 GHz or 24 cm wavelength) only permit a partial assessment of the grounding line position because the repeat pass cycle of the satellites is too long (35, 6 to 12, 24, and 46 d repeat, respectively) to maintain phase coherence over the fast-moving, main trunk. No high coherence DInSAR data could be obtained until the Agencia Spaziale Italiana (ASI) launched the Cosmo-SkyMed constellation (X-band, 9.65 GHz or 3.10 cm wavelength) in 2015. We used 4 × CSK 1-d repeat DInSAR pairs acquired 16 d apart in 2016 and 6 × DInSAR pairs acquired in 2017 to reveal that the grounding line of the main trunk migrated at tidal frequencies over several kilometers, while other sectors showed variable grounding line migration with data gaps in between (18). Data collection resumed in 2019 to 2021 with the launch of the ASI CSK constellation. Going forward, we use the ICEYE constellation to obtain a continuous picture of the grounding zone dynamics of Thwaites.

### Predictions of SSH.

We calculate changes in SSH using the CATS2008 tidal model (28) at a location off shore in front of Thwaites, corrected for IBE using ERA-5 atmospheric pressure fields at the same location (24). The agreement between DInSAR and SSH from IBE-corrected tide is excellent (0 mean with σ=8cm) (Fig. 3*B*).

### Hydraulic Potential.

quantifies the potential for water to move along the base of an ice sheet. We calculate overburden hydraulic potential $\phi =\rho_{i}gz_{s}+\left(\rho_{w}-\rho_{i}\right)gz_{b}$, where $\rho_{i}=$917 kg/m^3^ the density of ice, $\rho_{w}=$1,028 kg/m^3^, $z_{s}$ is the surface elevation of the ice sheet above mean sea level, and $z_{b}$ is the bed elevation above mean sea level. Because the multiplicative coefficient of $z_{b}$ is 10 × times less than for $z_{s}$, the gradients in hydraulic potential are often dominated by the gradients in ice surface elevation, yet bed slope is also larger than surface slope because glacier deformation over a bumpy bed only transmits very long wavelengths (10 to 20 ice thickness) in bed topography.

### Subglacial Hydrology.

is reproduced using the 2D finite-element Glacier Drainage System (GlaDS) model (30, 31), which predicts the presence of long, high-pressured subglacial channels and an adjacent distributed drainage system. GlaDS calculates water discharge, flux, and pressure beneath Thwaites Glacier. The model allows coincident development of distributed and channelized systems. We test a range of conductivity of the distributed and channelized systems to converge to a stable solution. We use outputs that produce a) an upper limit for water pressure, beyond which the model does not converge, b) a lower limit when solutions have water with pressures far below the overburden pressure, and c) intermediate levels of pressure. We present an output (c) with a distributed system conductivity of 1 × 10^−4^ m^3/2^ kg^−1/2^ and a channel conductivity of 5 × 10^−2^ m^3/2^ kg^−1/2^. The model is applied with basal sliding velocity and water production (geothermal and frictional heat) from the ISSM IMSIP-6 Antarctic control run along with surface and bed topographies from BedMachine Antarctica (47). The model is run on a mesh of 19,340 nodes with refining near the grounding line giving a minimum edge length of 280 m. The model is run for 20,000 d until near steady state. The equations for GlaDS are described in ref. 30 with the parameters shown in table 1 of ref. 48.

## Data Availability

Remote sensing data have been deposited in Dryad UCI (https://doi.org/10.5061/dryad.3ffbg79rm) (49).

## References

[1] E. Rignot , Four decades of Antarctic Ice Sheet mass balance from 1979 to 2017. Proc. Natl. Acad. Sci. U.S.A. **116**, 1095–1103 (2019). 30642972 10.1073/pnas.1812883116 PMC6347714

[2] P. R. Holland, S. L. Bevan, A. J. Luckman, Strong ocean melting feedback during the recent retreat of Thwaites Glacier. Geophy. Res. Lett. **50**, e2023GL103088 (2023).

[3] S. Schmidtko, K. J. Heywood, A. F. Thompson, S. Aoki, Multidecadal warming of Antarctic waters. Science **346**, 1227–1232 (2014). 25477461 10.1126/science.1256117

[4] N. J. Abram , Evolution of the Southern Annular Mode during the past millennium. Nat. Clim. Change **4**, 564–569 (2014).

[5] D. W. J. Thompson, S. Solomon, Interpretation of recent Southern Hemisphere climate change. Science **296**, 895–899 (2002). 11988571 10.1126/science.1069270

[6] P. R. Holland , Anthropogenic and internal drivers of wind changes over the Amundsen Sea, West Antarctica, during the 20th and 21st centuries. Cryosphere **16**, 5085–5105 (2022).

[7] H. Yu, E. Rignot, H. Seroussi, M. Morlighem, Retreat of Thwaites Glacier, West Antarctica, over the next 100 years using various ice flow models, ice shelf melt scenarios and basal friction laws. Cryosphere **12**, 3861–3876 (2018).

[8] R. H. Thomas, Force-perturbation analysis of recent thinning and acceleration of Jakobshavn Isbrae, Greenland. J. Glaciol. **50**, 57–66 (2004).

[9] E. Rignot, J. Mouginot, B. Scheuchl, Antarctic grounding line mapping from differential satellite radar interferometry. Geophys. Res. Lett. **38**, 1–6 (2011).

[10] W. Rack, M. King, O. J. Marsh, C. T. Wild, D. Floricioiu, Analysis of ice shelf flexure and its InSAR representation in the grounding zone of the southern McMurdo Ice Shelf. Cryosphere **11**, 2481–2490 (2017).

[11] C. T. Wild, O. Marsh, W. Rack, Differential interferometric synthetic aperture radar for tide modelling in Antarctic ice-shelf grounding zones. Cryosphere **13**, 3171–3191 (2019).

[12] P. Friedl, F. Weiser, A. Fluhrer, M. H. Braun, Remote sensing of glacier and ice sheet grounding lines: A review. Earth-Sci. Rev. **201**, 102948 (2020).

[13] Y. Mohajerani , Automatic delineation of glacier grounding lines in differential interferometric synthetic-aperture radar data using deep learning. Nat. Sci. Rep. **11**, 4992 (2021). 10.1038/s41598-021-84309-3 PMC7925556 33654148

[14] V. Ignatenko *et al*., “ICEYE microsatellite SAR constellation status update: Evaluations of first commercial imaging modes” in *IGARSS 2020—2020 IEEE International Geoscience and Remote Sensing Symposium, Waikoloa, HI* (2020).

[15] M. Morlighem , Deep glacial troughs and stabilizing ridges unveiled beneath the margins of the Antarctic ice sheet. Nat. Geosci. **13**, 132–137 (2020).

[16] E. Rignot, J. Mouginot, M. Morlighem, H. Seroussi, B. Scheuchl, Widespread, rapid grounding line retreat of Pine Island, Thwaites, Smith, and Kohler Glaciers, West Antarctica, from 1992 to 2011. Geophys. Res. Lett. **41**, 3502–3509 (2014).

[17] I. Joughin, B. E. Smith, B. Medley, Marine ice sheet collapse potentially under way for the Thwaites Glacier Basin, West Antarctica. Science **344**, 735–738 (2014). 24821948 10.1126/science.1249055

[18] P. Milillo , Heterogeneous retreat and ice melt of Thwaites Glacier, West Antarctica. Sci. Adv. **5**, eaau3433 (2019). 30729155 10.1126/sciadv.aau3433 PMC6353628

[19] N. Golledge , Antarctic climate and ice-sheet configuration during the early Pliocene interglacial at 4.23 Ma. Clim. Past **13**, 959–975 (2017).

[20] E. Rignot, Evidence for rapid retreat and mass loss of Thwaites Glacier, West Antarctica. J. Glaciol. **47**, 213–222 (2001).

[21] C. T. Wild , Weakening of the pinning point buttressing Thwaites Glacier, West Antarctica. Cryosphere **16**, 397–417 (2022).

[22] G. Gudmundsson, J. Barnes, D. Goldberg, M. Morlighem, Limited impact of Thwaites Ice Shelf on future ice loss from Antarctica. Geophys. Res. Lett. **50**, e2023GL102880 (2023).

[23] P. Milillo , On the short-term grounding zone dynamics of Pine Island Glacier, West Antarctica, observed with COSMO-SkyMed interferometric data. Geophys. Res. Lett. **44**, 10436–10444 (2017).

[24] H. Chen, E. Rignot, B. Scheuchl, S. Ehrenfeucht, Grounding zone of Amery Ice Shelf, Antarctica, from differential synthetic-aperture radar interferometry. Geophys. Res. Lett. **50**, e2022GL102430 (2023).

[25] W. Brancato , Grounding line retreat of Denman Glacier, East Antarctica, measured with COSMO-SkyMed radar interferometry data. Geophys. Res. Lett. **47**, e2019GL086291 (2020).

[26] A. Chartran, I. M. Howat, A comparison of contemporaneous airborne altimetry and ice-thickness measurements of Antarctic ice shelves. J. Glaciol. **49**, 1–14 (2023).

[27] K. Brunt, H. A. Fricker, L. Padman, T. Scambos, S O’neel,, Mapping the grounding zone of the Ross Ice Shelf, Antarctica, using ICESat laser altimetry. Ann. Glaciol. **51**, 71–79 (2010).

[28] L. Padman, S. Y. Erofeeva, H. A. Fricker, Improving Antarctic tide models by assimilation of ICESat laser altimetry over ice shelves. Geophy. Res. Lett. **35**, L22504 (2008).

[29] J. A. MacGregor , The scientific legacy of NASA’s operation IceBridge. Rev. Geophys. **59**, e2020RG000712 (2021).

[30] M. Werder, I. Hewitt, C. Schoof, G. Flowers, Modeling channelized and distributed subglacial drainage in two dimensions. J. Geophys. Res. Earth Surf. **118**, 2140–2158 (2013).

[31] C. Dow, The role of subglacial hydrology in Antarctic ice sheet dynamics and stability: A modelling perspective. Ann. Glaciol. **9**, 1–6 (2023).

[32] R. T. Walker , Ice-shelf tidal flexure and subglacial pressure variations. Earth Planet Sci. Lett. **361**, 422–428 (2013).

[33] E. Wilson, A. J. Wells, I. J. Hewitt, C. Cenedese, The dynamics of a subglacial salt wedge. J. Fluid Mech. **895**, A20 (2020).

[34] K. Warburton, D. Hewitt, J. Neufeld, Tidal grounding-line migration modulated by subglacial hydrology. Geophy. Res. Lett. **47**, e2020GL089088 (2020).

[35] M. Heinert, B. Riedel, Parametric modelling of the geometrical ice–ocean interaction in the Ekstroemisen grounding zone based on short time-series. Geophys. J. Int. **169**, 407–420 (2007).

[36] D. M. Holland, K. W. Nicholls, A. Basinski, The southern ocean and its interaction with the Antarctic ice sheet. Science **367**, 1326–1330 (2020). 32193320 10.1126/science.aaz5491

[37] A. Wåhlin , Pathways and modification of warm water flowing beneath Thwaites Ice Shelf, West Antarctica. Sci. Adv. **7**, eabd7524 (2021). 10.1126/sciadv.abd7254 PMC8034858 33837074

[38] L. Gray , Evidence for subglacial water transport in the West Antarctic Ice Sheet through three-dimensional satellite radar interferometry. Geophys. Res. Lett. **32**, L03501 (2005).

[39] B. P. Parizek , Dynamic (in)stability of Thwaites Glacier, West Antarctica. J. Geophys. Res. Earth Surf. **118**, 1–18 (2013).

[40] H. Seroussi, M. Morlighem, Representation of basal melting at the grounding line in ice flow models. Cryosphere **12**, 3085–3096 (2018).

[41] A. Robel, E. Wilson, H. Seroussi, Layered seawater intrusion and melt under grounded ice. Cryosphere **16**, 451–469 (2022).

[42] W. Chu , Multisystem synthesis of radar sounding observations of the Amundsen Sea sector from the 2004–2005 field season. J. Geophys. Res. Surf. **126**, e2021JF006296 (2021). 10.1029/2021JF006296 PMC9286636 35865452

[43] C. Burgard, N. C. Jourdain, R. Reese, A. Jenkins, P. Mathiot, An assessment of basal melt parameterisations for Antarctic ice shelves. Cryosphere **16**, 4931–4975 (2022).

[44] S. Koellner, B. R. Parizek, R. B. Alley, A. Muto, N. Holschuh, The impact of spatially-variable basal properties on outlet glacier flow. Earth Planet Sci. Lett. **515**, 200–208 (2019).

[45] A. Aschwanden, T. Bartholomaus, D. Brinkerhoff, M. Truffer, Brief communication: A roadmap towards credible projections of ice sheet contribution to sea level. Cryosphere **15**, 5705–5715 (2021).

[46] P. Milillo , Rapid glacier retreat rates observed in West Antarctica. Nat. Geosci. **15**, 48–53 (2022).

[47] H. Seroussi , initMIP-Antarctica: An ice sheet model initialization experiment of ISMIP6. Cryosphere **13**, 1441–1471 (2019).

[48] C. Dow , Totten Glacier subglacial hydrology determined from geophysics and modeling. Earth Planet. Sci. Lett. **531**, 115961 (2020).

[49] E. Rignot, Widespread seawater intrusions beneath the grounded ice of Thwaites Glacier, West Antarctica [Dataset]. Dryad. 10.5061/dryad.3ffbg79rm. Deposited 19 March 2024. PMC11145208 38768351

